# Bone Mineral Density Assessment by Dual-Energy X-Ray Absorptiometry (DEXA) Versus Serum Tartrate-Resistant Acid Phosphatase 5b (TRAP-5b) in Children With Classic Salt-Losing Congenital Adrenal Hyperplasia

**DOI:** 10.7759/cureus.83778

**Published:** 2025-05-09

**Authors:** Noura M ElBakry, Asmaa Khalf, Mohamed Ameen, Mohamed Mahgoob

**Affiliations:** 1 Pediatric, Faculty of Medicine, Minia University, Minia, EGY; 2 Clinical Pathology, Faculty of Medicine, Minia University, Minia, EGY; 3 Radiology, Faculty of Medicine, Minia University, Minia, EGY; 4 Pediatrics, Faculty of Medicine, Minia University, Minia, EGY

**Keywords:** bone mineral density, cah, congenital adrenal hyperplasia, dexa, trap-5b

## Abstract

Chronic glucocorticoid (cGC) therapy is a leading cause of drug-induced osteoporosis, often resulting in severe bone loss. Bone mineral density (BMD) is maintained by a dynamic balance between bone formation and resorption, regulated by various hormones, vitamin D, and cytokines. BMD is typically measured using dual-energy X-ray absorptiometry (DEXA). Tartrate-resistant acid phosphatase 5b (TRAP-5b) serves as a biomarker of bone resorption and is utilized to assess osteoclast activity. Objectives: This study aimed to evaluate BMD by measuring serum TRAP-5b levels in patients with congenital adrenal hyperplasia (CAH) receiving steroid therapy. Methods: Forty patients with CAH underwent DEXA scans to assess BMD. Patients were then divided into two groups: one with normal BMD (n=14) and the other with abnormal BMD (n=26). Serum TRAP-5b levels were measured as a marker of bone turnover. Results: Children with CAH had significantly lower BMD in both the vertebrae and femoral neck. TRAP-5b levels were significantly higher in the abnormal BMD group (2.95 U/L) compared to the normal BMD group (p<0.001), indicating increased bone resorption in CAH. Conclusions: Serum TRAP-5b activity may be a significant diagnostic and prognostic marker for bone disease in CAH.

## Introduction

Congenital adrenal hyperplasia (CAH) is an autosomal recessive disorder characterized by inadequate synthesis of adrenal glucocorticoids, frequently associated with impaired mineralocorticoid production. In classic CAH, approximately 90% of cases are attributable to 21-hydroxylase deficiency, which is a key enzyme involved in the conversion of progesterone to deoxycorticosterone and 17-hydroxyprogesterone to 11-deoxycortisol. This deficiency disrupts the hypothalamic-pituitary-adrenal axis feedback loop, leading to adrenal gland hyperplasia. Consequently, cortisol and aldosterone precursors are diverted toward androgen production, leading to virilization and chronic hyperandrogenism [1]. The prevalence of CAH in Egypt is reported to be tenfold higher than the global average [2].

Children with CAH require lifelong glucocorticoid replacement therapy to compensate for deficient cortisol and suppress excessive adrenal androgen production. Maintaining hormonal balance throughout childhood is crucial for optimal growth and minimizing hyperandrogenism [3]. However, achieving complete suppression of androgen precursors without causing iatrogenic Cushing's syndrome, which may impede growth and development, presents significant challenges [4].

Extended high-dose glucocorticoid administration adversely affects bone mineral density (BMD) via mechanisms including reduced osteoblast activity, decreased gastrointestinal calcium absorption, increased renal calcium loss, and enhanced bone resorption [5]. Conversely, excess androgens can protect BMD by stimulating osteoblast activity [6].

Dual-energy X-ray absorptiometry (DXA) is widely recognized as a valuable tool for assessing bone health and fracture risk, particularly in adults [7]. Its application in pediatric populations has significantly increased over the past 10-15 years due to the development of appropriate age reference data from healthy children [8]. When combined with fracture history, DXA results can contribute significantly to osteoporosis diagnosis in children and adolescents [9].

Tartrate-resistant acid phosphatase (TRAP), a class of metalloenzymes, is expressed by osteoclasts, macrophages, and dendritic cells [10]. TRAP-5b, an osteoclast-produced isoform, is considered a reliable marker of bone resorption [11]. TRAP-5b levels correlate inversely with BMD in postmenopausal women and have been proposed as predictors of hip or vertebral fracture risk [12]. TRAP-5b levels are unaffected by kidney or liver dysfunction, exhibit no circadian variation, and are not influenced by dietary factors, thus serving as a reliable marker for assessing osteoporosis treatment and predicting future fracture risk [9].

This study aims to examine TRAP-5b as a bone turnover marker and its relationship with BMD in children with classic CAH, addressing the inconsistent results of prior studies on BMD and bone turnover markers in this population.

## Materials and methods

This cross-sectional study recruited children with classic salt-losing CAH who were regularly followed at the Pediatric Endocrinology Outpatient Clinic of Minia University Children's Hospital, Egypt. Participants were randomly selected between January 2023 and February 2024. They were divided into two groups based on their bone mineral density (BMD) as measured by DEXA scan: Group I: Fourteen patients with normal bone density; Group II: Twenty-six patients with abnormal bone density (Table 1).

**Table 1:** Inclusion and exclusion criteria of the study groups CAH: Congenital adrenal hyperplasia, BMD: Bone mineral density

Inclusion criteria	Exclusion criteria
Children included were diagnosed with classic salt-losing CAH confirmed by medical records	Children excluded were those with other conditions that could affect BMD assessment
History of salt-wasting crisis	Other endocrine problems
Documented low serum sodium	Cardiac, hepatic, or renal diseases
Hyperkalemia	Documented fractures of the vertebrae, wrist, or hip
Markedly elevated 17-hydroxyprogesterone (17-OHP) and plasma renin activity levels	Use of medications that might affect bone metabolism: Vitamin D preparations Calcium supplements Gonadotropin-releasing hormone analogues

Prior to enrollment, the study's objectives and procedures were explained to each participant's parent or legal guardian. Written informed consent was obtained from all parents/guardians, who were assured of their right to withdraw from the study at any time without penalty. No deceptive practices were used. The Pediatric Department Council and the Institutional Review Board, Faculty of Medicine, Minia University, approved the study protocol "MUFMIRB" (approval number: 1129/04/2024).

Participants underwent a comprehensive evaluation including the following: Detailed medical history, physical examination, anthropometric measurements (height, weight), and laboratory investigations (calcium [Ca], phosphorus [Ph], alkaline phosphatase [ALP], 17-OHP, and adrenocorticotropic hormone [ACTH]). Serum tartrate-resistant acid phosphatase type 5 (TRAP-5b) levels were measured utilizing enzyme-linked immunosorbent assay (ELISA) using Bioassay Technology Laboratory ELISA, Changsheng S Rd, Nanhu Dist, Jiaxing, Zhejiang, China (catalog # E7393Hu). The optical density was determined at 450 nm using a microplate reader (Huma Reader 3700, Germany).

Height and weight were measured three times on a calibrated stadiometer on the day of the DXA exam and averaged. Height and weight Z-scores were calculated using Gene CALC 3.0 software (National Center for Biotechnology Information, Bethesda, Maryland).

Hydrocortisone (HC) served as the primary glucocorticoid for 79% of patients; doses of prednisone and dexamethasone administered to the remaining patients were converted to equivalent HC doses (mg/m²/day). The mean HC dosage was determined based on clinic visit records from 3, 6, and 12 months before the DEXA scan. Bone status, including bone mineral content (BMC) and bone mineral density (BMD), was evaluated using DEXA at the lumbar spine and femoral neck, with results converted to age- and sex-matched Z-scores [6].

BMD was classified as follows: Osteopenia: Z-scores between -1 and -2.5, Osteoporosis: Z-scores below -2.5, Normal BMD: Z-score > -1 SD, Low BMD: Z-score < -1 SD [4]. Total body bone mineral density (TBMD) Z-scores were calculated using pediatric software and adjusted for height-for-age Z-scores (TBMDHAZ) [6].

Key details regarding the DEXA scans: DEXA scans were performed within three months of obtaining radiographs. All patients followed the same standardized pediatric protocol for preparation, positioning, and image acquisition [2]. Patient data (name, birth date, weight, height, and bone age) were entered into the software (GE Lunar enCORE v.11.40.004 [GE Healthcare, Chicago, USA]).

A professional member of the International Society for Clinical Densitometry (ISCD) evaluated the DEXA scans, considering both chronological and bone ages. The software automatically switched between chronological and bone age as reported, ensuring that bone mass assessment was appropriate for the corresponding age [6]. BMD assessment included total body (excluding the head) and lumbar spine (L1-L4) densitometry [7,8]. DEXA scans were conducted at each clinical center utilizing one densitometer, specifically Hologic, Inc. (Bedford, MA) models, including QDR4500A, QDR4500W, Delphi A, and Apex. Software versions for acquisition ranged from 11.1 to 12.7 (Apex 2.1). Scans were obtained according to the manufacturer's guidelines for patient positioning. Whole-body, posterior-anterior lumbar spine (L1-L4, fast array), non-dominant forearm, and left proximal femur (fast array) scans were obtained for each participant. Cross-calibration of DEXA devices and longitudinal calibration stability were monitored as previously described [9-11]. All scans were centrally analyzed by the DEXA Core Laboratory (University of California, San Francisco, San Francisco, CA) using Hologic software version Discovery 12.3 for baseline scans.

**Figure 1:**
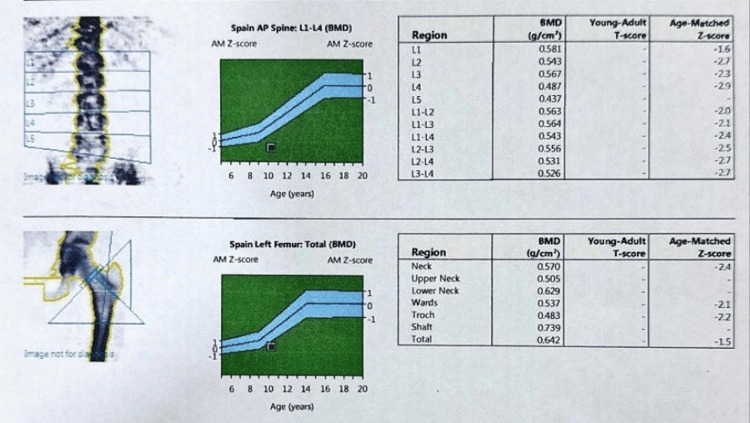
DEXA scan report showing low bone mineral density in a 10-year-old female

A 10-year-old girl's DEXA scan of her lumbar spine (L1-L4) and left proximal femur indicates reduced bone mineral density compared to what's typical for her age. Specifically, the age-matched Z-scores for her lumbar vertebrae range from -1.6 to -2.9, resulting in a composite Z-score of -2.4. All of these values fall below the International Society for Clinical Densitometry (ISCD) pediatric threshold for "low bone mineral density for chronological age (Figure 1).

Sample size calculations were performed using GRANMO (2012) software (dataRus, Barcelona, Spain), with an alpha risk of 0.05 and a beta risk of 0.2 in a two-sided test. Data are presented as means ± standard deviation (SD). The Shapiro-Wilk test was used to assess data normality, while Levene's test evaluated the homogeneity of variances. For normally distributed continuous variables, comparisons were made using the Student's t-test for unpaired samples; for non-normally distributed data, the Mann-Whitney U test was applied. Categorical data were analyzed using the chi-squared (χ²) test or Fisher's exact test as appropriate. Pearson's correlation coefficient (rho) was used to assess associations between variables. All tests were two-tailed, with statistical significance defined as p < 0.05. All statistical analyses were performed using IBM Corp. Released 2020. IBM SPSS Statistics for Windows, Version 27.0. Armonk, NY: IBM Corp.

Children with CAH in the Minia Governorate, Egypt, are typically treated at the pediatric endocrinology clinic of Minia University Tertiary Hospital, as it is the only specialized center providing care for this condition in the region.

## Results

The CAH and control groups were effectively matched for age (p = 0.74) and sex (p = 0.37), minimizing the influence of these variables on subsequent measurements. However, significant differences were observed in growth parameters. Children with CAH exhibited significantly lower height-for-age z-scores (HAZ) (p < 0.001), indicating shorter stature compared to their healthy peers, while they had significantly higher weight-for-age (WAZ) (p = 0.006) and body mass index (BAZ) z-scores (p < 0.001), suggesting a greater propensity for overweight or obesity (Table 2).

**Table 2:** Clinical characteristics of the studied groups * Significant <0.05, HAZ: Height z score, WAZ: Weight z score, BAZ: Body mass index z score, CAH: Congenital adrenal hyperplasia, IQR: Interquartile range, SD: Standard deviation

	CAH children (N=40)	Controls (n=40)	P-value
Age (years)			
Median (IQR)	5.3 (5-6)	5.4 (5-6)	0.74
(mean ± SD)	5.8 ± 1.04	5.9 ± 1.01	
Sex n (%)			
Male	22 (55%)	18 (45%)	0.37
Female	18 (45%)	22 (55%)	
Height z score (HAZ) (mean ± SD)	-1.96 ± 1.9	0.03 ± 1.6	<0.001*
Weight z score (WAZ) (mean ± SD)	1.31 ± 1.19	0.62±0.96	0.006*
BMI z score (BAZ) (mean ± SD)	2.26±1.16	0.77±1.18	<0.001*

CAH children had significantly lower BMD at both the lumbar spine and femoral neck (p<0.001), independent of height, as shown by lower BMD, BMD Z-scores, and BMDHAZ. They also had elevated TRAP-5b levels (p<0.001), indicating increased bone resorption and an imbalance in bone remodeling (Table 3).

**Table 3:** DEXA scanning parameters & serum TRAP- 5b levels in CAH children and controls * Significant <0.05, CAH: Congenital adrenal hyperplasia, IQR: Interquartile range, SD: standard deviation

	CAH children (N=40)	Controls (N=40)	P-value
Lumbar spine (L1-L4) BMD [g/cm2] (mean ± SD)	0.68±0.31	0.91±0.18	<0.001*
BMD Z-scores for Lumbar spine (mean ± SD)	2.3±4.9	6.1±3.5	<0.001*
BMD_HAZ_ Z-scores for Lumbar spine (mean ± SD)	3.3±5.2	7.2±3.3	<0.001*
Femoral neck BMD [g/cm2] (mean ± SD)	0.73±0.30	0.95±0.13	<0.001*
BMD Z-scores for femoral neck (mean ± SD)	2.8±6.7	7.8±5.5	<0.001*
BMD_HAZ_ Z-scores for femoral neck (mean ± SD)	3.6±6.8	8.4±5.4	<0.001*
TRAP- 5b (ng/ml) Median (IQR) (mean ± SD)	1.2 (1-2.8) 1.8± 1.09	0.40(0.22-0.80) 0.49± 0.27	<0.001*

Children with abnormal BMD received higher glucocorticoid doses (26.1 vs. 14.3 mg/m²/day, p<0.001) and had poorer CAH control (43.8% vs. 4.2%, p=0.001) than those with normal BMD. These factors, not age, sex, disease duration, or other growth parameters, are key determinants of bone health in CAH children (Table 4).

**Table 4:** Clinical characteristics of CAH children with normal and abnormal BMD * Significant <0.05, BMD: Bone mineral density, CAH: Congenital adrenal hyperplasia, IQR: Interquartile range, SD: Standard deviation

	Group I (Normal BMD) (N=24)	Group II (Abnormal BMD) (N=16)	P-value
Age (years)			0.16
Median (IQR)	5(5-5.6)	5.8(5.2-7.2)	
(mean ± SD)	5.5± 0.92	6.1± 1.1	
Sex n (%)			0.89
Male	11(45.8 %)	7(43.8%)	
Female	13 (54.2%)	9(56.3%)	
Age at Diagnosis (Months)			0.09
Median (IQR)	3(3-3.6)	3.8(3.2-5.3)	
(mean ± SD)	3.5± 0.92	4.1± 1.1	
Disease Duration (years) (mean ± SD)	4.5± 1.5	5± 1.6	0.23
Dose of Glucocorticoids [Mg/m2/Day] (mean ± SD)	14.3± 5	26.1± 3.4	<0.001*
Dose of mineralocorticoid [mg/day] (mean ± SD)	0.15± 0.05	0.15± 0.05	
Well control	23(95.8%)	9(56.3%)	0.001*
Poor control	1(4.2%)	7(43.8%)	
Height z score (mean ± SD)	-1.6±2.1	-1.7±1.7	0.97
Weight z score (mean ± SD)	1.4±1.4	1.2±0.73	0.52
BMI z score (mean ± SD)	2.66±1.26	2.16±0.95	0.18

The abnormal BMD group had lower vitamin D and calcium, along with higher alkaline phosphatase, ACTH, 17-OH progesterone, androstenedione, and TRAP-5b (p<0.05), indicating increased bone turnover and hormonal imbalances. As expected, this group also had lower BMD, BMD Z-scores, and BMDHAZ (Table 5).

**Table 5:** Laboratory data in the studied CAH children with normal and abnormal BMD * Significant, WAZ: Weight z score, HAZ: Height z score, BAZ: Body mass index z score, CAH: Congenital adrenal hyperplasia, BMD: Bone mineral density, ACTH: Adrenocorticotropic, SD: Standard deviation, DHEA: Dehydroepiandrosterone, TRAP-5b: Tartrate resistant acid phosphatase-5b, 17-OHP: 17-hydroxyprogesterone.

	Group I (Normal BMD) (N=14)	Group II (Abnormal BMD) (N=26)	P-value
Vitamin D(IU) Median (IQR) (mean ± SD)	34.2(30.3-43.4) 34.7±12.8	13(10.4-15.8) 16.5±13.4	<0.001*
Calcium (mg/dl) Median (IQR) (mean ± SD)	1.2(0.88-1.9) 1.5±0.30	0.95(0.90-1.1) 0.98±0.28	0.01*
Alkaline phosphatase (U/L) Median (IQR) (mean ± SD)	124(50.6-144) 139±178	248(189-336) 253±129	<0.001*
ACTH (am) (pg/ml) Median (IQR) (mean ± SD)	38.6(32.5-44) 66.7±173	49.1(43.4-72.6) 64.3±36.6	0.001*
Cortisol (am) (mcg/dl) Median (IQR) (mean ± SD)	12.6(11.7-132.2) 12±5.07	13.2(8-21.2) 13.2±8.2	0.19
17 OHP (ng/ml) Median (IQR) (mean ± SD)	3.1(2.6-3.4) 3±0.49	3.6 (3-24.8) 12.9±13.7	0.001*
Androstenedione (ng/dl) Median (IQR) (mean ± SD)	0.80(0.47-3.5) 8.2±16.2	30.6(22.6-43.4) 33.2±20.4	0.001*
DHEA (ng/dl) Median (IQR) (mean ± SD)	3.2(2.4-3.5) 4.7±5.5	3.5(2.4-22.8) 12±13.1	0.10
TRAP- 5b(ng/mL) Median (IQR) (mean ± SD)	1.1(0.90-1.2) 1.12±0.40	2.95(2.3-3.4) 2.95±0.82	<0.001*
Lumbar spine (L1-L4) BMD [g/cm2] (mean ± SD)	0.92±0.13	0.35±0.15	<0.001*
BMD Z-scores for Lumbar spine (mean ± SD)	6.3±2.6	-3.1±1.6	<0.001*
BMD_HAZ_ Z-scores for Lumbar spine (mean ± SD)	7.5±2.4	-2.3±2.5	<0.001*
Femoral neck BMD [g/cm2] (mean ± SD)	0.95±0.13	0.43±0.13	<0.001*
BMD Z-scores for femoral neck (mean ± SD)	7.8±5.1	-3.3± 2.09	<0.001*
BMD_HAZ_ Z-scores for femoral neck (mean ± SD)	8.6±4.9	-2.8±2.6	<0.001*

Correlation analysis revealed that TRAP-5b levels were strongly correlated with glucocorticoid dose (r = 0.66, p < 0.001), vitamin D (r = -0.66, p < 0.001), alkaline phosphatase (r = 0.74, p < 0.001), and androstenedione (r = 0.69, p < 0.001), and negatively correlated with femoral neck and lumbar spine BMD (r = -0.77, p < 0.001). TRAP-5b also demonstrated a moderate positive correlation with poor CAH control (r = 0.48, p = 0.002). No significant correlations were observed with age, disease duration, cortisol levels, mineralocorticoid dose, or growth parameters (Table 6).

**Table 6:** Correlations of serum TRAP- 5b with different parameters among CAH children * Significant <0.05, DHEA: Dehydroepiandrosterone, WAZ: Weight z score, HAZ: Height z score, BAZ: Body mass index z score, BMD: Bone mineral density, ACTH: Adrenocorticotropic, SD: Standard deviation

TRAP- 5b(ng/m)	R	P-value
Age (years)	0.23	0.14
Disease duration	0.17	0.26
Cortisol(am) (mcg/dl)	0.04	0.78
Dose of glucocorticoid (mg/m^2/^day)	0.66	<0.001*
Dose of mineralocorticoid (mg/day)	0.04	0.76
ACTH (am) (ng/dl)	0.28	0.07
Vitamin D(U/L)	-0.66	<0.001*
Alkaline phosphatase (IU/L)	0.74	<0.001*
17-hydroxy progesterone(ng/ml)	0.25	0.11
Androstenedione (ng/dL)	0.69	<0.001*
DHEA (ng/dL)	0.20	0.20
Poor control	0.48	0.002*
HAZ	0.10	0.53
WAZ	-0.02	0.86
BAZ	-0.11	0.49
Femur neck BMD	-0.77	<0.001*
Lumbar spine BMD	-0.77	<0.001*

Receiver operating characteristic (ROC) analysis showed that TRAP-5b has strong potential for identifying abnormal bone density in CAH children, with an area under the curve (AUC) of 0.90 (95% CI: 0.78-0.99, p < 0.001). At a cutoff value of >1.5 ng/mL, TRAP-5b demonstrated high sensitivity (89%), specificity (93.5%), positive predictive value (PPV) (80%), negative predictive value (NPV) (96%), and accuracy (92.5%), making it a valuable marker for assessing bone health in these children (Table 7).

**Table 7:** ROC curve analysis of TRAP- 5b level for diagnosis of abnormal bone density * Significant <0.05, AUC: Area under the curve, PPV: Positive predictive value, NPV: Negative predictive value

	TRAP- 5b
Optimal cutoff point	>1.5
AUC	0.90
95% CI	0.78-0.99
P value	<0.001*
Sensitivity	89%
Specificity	93.5%
PPV	80%
NPV	96%
Accuracy	92.5%

## Discussion

This study investigated bone mineral density (BMD) and related biochemical markers in children with congenital adrenal hyperplasia (CAH), finding a high prevalence of osteopenia (42%) and osteoporosis (23%) compared to only 35% with normal BMD, a finding consistent with other reports [10]. This high prevalence underscores the importance of monitoring bone health in this population. The study observed lower BMD in CAH children compared to controls, a finding corroborated by some [11] but not all previous research [2,4]. Similarly, the study's findings regarding alkaline phosphatase and vitamin D levels were consistent with some studies [13] but differed from others [14]. These discrepancies may arise from variations in patient characteristics, including age, pubertal stage, steroid type and dose, CAH subtype, and methods for controlling androgen exposure.

A key finding of the study was the significant elevation of serum TRAP-5b, a marker of bone resorption, in the abnormal BMD group (p < 0.001), indicating increased bone resorption, which aligns with previous research [15]. A significant correlation was found between TRAP-5b and glucocorticoid dose (p < 0.001), supporting the established link between steroid therapy and altered bone metabolism [16]. TRAP-5b also correlated with androstenedione levels (p < 0.001), consistent with the association between low androgens and low bone mass [4]. Additionally, significant correlations were observed between TRAP-5b and 17OHP and BMI (p < 0.05, 0.024, and <0.001, respectively), potentially reflecting higher steroid doses in poorly controlled and overweight patients.

The study explored the potential mechanisms contributing to altered bone metabolism in CAH. In congenital adrenal hyperplasia (CAH), chronic glucocorticoid therapy is essential to replace deficient cortisol and suppress androgen overproduction. However, this treatment can contribute to a high bone turnover rate in children with CAH, due to the complex interaction between exogenous glucocorticoids and endogenous androgens [16-18]. Long-term glucocorticoid use is a recognized risk factor for osteoporosis [19-24]. It is widely understood that glucocorticoids inhibit bone formation, primarily by affecting osteoblast development and osteocyte survival, and simultaneously stimulate bone resorption through increased osteoclast activity [25-28]. This dual action leads to rapid bone loss. Furthermore, glucocorticoids can disrupt skeletal growth factors like insulin-like growth factor-I (IGF-I), further impacting bone health.

The study divided children into groups based on DEXA BMD (normal and abnormal). While some studies have reported gender differences in BMD [4], this study did not find such a difference. However, it did observe a higher average steroid dose in the abnormal BMD group, supporting previous findings that higher hydrocortisone equivalent doses (>20 mg/m²/day) correlate with lower lumbar BMD Z-scores [23]. Elevated 17-OHP levels (mean ± SD: 12.9 ± 13.7) were significantly associated with abnormal BMD (p < 0.001), indicating that poorly controlled patients (17-OHP >10 nmol/L) experienced greater bone density reduction, consistent with other reports [11]. Higher DHEAS and androstenedione levels were also linked to abnormal BMD, which contrasts with studies suggesting a protective effect of DHEA on bone [2].

The study suggests that monitoring serum TRAP-5b activity could serve as a more cost-effective and less invasive alternative to DEXA scanning for monitoring BMD in CAH children due to its lower cost and less invasive nature.

Limitations of the study

The cross-sectional design of the study limits the establishment of causality between observed associations, necessitating longitudinal studies to determine temporal relationships. Variability in glucocorticoid regimens, despite conversion to hydrocortisone equivalents, and the lack of standardized timing of dose administration relative to DEXA scans may have introduced variability. Furthermore, the study's focus primarily on glucocorticoids, androgens, and TRAP-5b meant that other important bone health factors like calcium intake, vitamin D status beyond serum levels, physical activity, and genetic predisposition were not comprehensively assessed. The potential single-center nature of the study could also limit the generalizability of the findings, and despite cross-calibration efforts, the use of different DEXA scanners and software versions across centers might have introduced measurement variability.

## Conclusions

The study identified a strong association between higher glucocorticoid doses and abnormal bone mineral density (BMD), highlighting the importance of careful dose management. The group with abnormal BMD also showed significantly elevated levels of 17-OHP, indicating the negative impact of poor hormonal control on bone health. Furthermore, significant correlations were found between higher levels of DHEAS and androstenedione and abnormal BMD, suggesting a complex role of adrenal androgens in bone homeostasis. Serum TRAP-5b emerged as a valuable marker of bone resorption, demonstrating a strong correlation with abnormal BMD, glucocorticoid dose, 17-OHP, and BMI, with ROC curve analysis further validating its efficacy in identifying abnormal bone density.

These findings suggest that TRAP-5b could serve as a useful tool for monitoring bone health in children with CAH, potentially offering a less invasive and more cost-effective alternative to DEXA scanning. The study also reinforces the critical importance of precise glucocorticoid dosing and strict hormonal control to mitigate the risk of bone complications in this vulnerable population. Further research is warranted to explore the long-term effects of CAH and its treatment on bone health and to optimize monitoring and intervention strategies.
